# Feasibility of non-contrast-enhanced four dimensional (4D) MRA in head and neck tumors, comparison with contrast-enhanced 4D MRA

**DOI:** 10.1186/s40064-016-2953-3

**Published:** 2016-08-08

**Authors:** Mio Sakai, Till Illies, Nadine Jerusel, Souichirou Tateishi, Masato Uchikoshi, Jens Fiehler, Yoshiyuki Watanabe, Katsuyuki Nakanishi, Noriyuki Tomiyama

**Affiliations:** 1 Department of Diagnostic Radiology, Osaka Medical Center for Cancer and Cardiovascular Diseases, 1-3-3 Nakamichi, Higashinari-ku, Osaka 537-8511 Japan; 2 Department of Diagnostic and Interventional Neuroradiology, University Medical Center Hamburg-Eppendorf, Hamburg, Germany; 3 Siemens Healthcare GmbH, Erlangen, Germany; 4 Department of Diagnostis and Interventional Radiology, Graduate School of Medicine, Osaka University, Osaka, Japan

**Keywords:** Non-contrast-enhanced MRA, Time-resolved MRA, Head and neck tumor, Hemodynamics

## Abstract

**Background:**

Information of tumor vascular architecture and hemodynamics is important in treating patients with head and neck tumors (HNTs). The purpose of this study is to investigate the feasibility of non-contrast-enhanced four-dimensional magnetic resonance angiography (non-CE 4DMRA) using arterial spin labeling for anatomical and hemodynamic evaluation of vascularity of head and neck tumors.

**Results:**

Non-CE 4DMRA images of 15 patients with HNTs were compared with those of contrast-enhanced 4DMRA (CE 4DMRA) by two independent observers. For qualitative evaluation, overall image quality, visualization of arterial branches and main arterial tumor feeders were assessed. For hemodynamic evaluation, signal-intensity-over-time curves within the tumors were compared. The sensitivity of non-CE 4DMRA for the identification of arterial branches and the main arterial tumor feeders was 75 and 20 %, respectively (interobserver agreement, κ = 0.56 and 0.54, respectively), while that of CE 4DMRA was 99 and 95 %, respectively (interobserver agreement, κ = 0.62 and 0.70, respectively). All three arterial/hypervascularized tumors determined on CE 4DMRA showed distinct signal-intensity-over-time curve pattern on non-CE 4DMRA, with distinct peak and wash out phases. Other tumors showed no wash out on non-CE 4DMRA.

**Conclusions:**

Use of non-CE 4DMRA for the anatomical and hemodynamic evaluation of vascularity of head and neck tumors is feasible, although the technique needs to be improved.

## Background

Information on tumor vascular architecture and hemodynamics is important in the management of patients with head and neck tumors (HNTs). The information helps in differential diagnosis and is a key input for treatment decision-making and planning for intravascular procedures such as preoperative embolization and/or intraarterial chemotherapy (Michaely et al. 2007; Nishimura et al. 2012; Lazzaro et al. 2011).

Digital subtraction angiography (DSA), the current gold standard for the assessment of HNT vasculature, is associated with a risk for complications, such as stroke due to embolism, involves radiation exposure, and requires injection of iodinated contrast material (Bi et al. 2010). Contrast-enhanced (CE) four-dimensional (4D) MRA (CE 4DMRA), i.e., time-resolved contrast-enhanced 3D MRA is reported to be a powerful and less invasive tool (Michaely et al. 2007; Nishimura et al. 2012). CE 4DMRA provides a 3D dynamic dataset with a sub-millimeter spatial resolution and a temporal resolution of a few seconds (Nishimura et al. 2012). However, the reliability of CE 4DMRA for the identification of feeding arteries is yet to be established (Nishimura et al. 2012). Furthermore, the procedure entails a risk of nephrogenic systemic fibrosis due to the use of gadolinium-based contrast agents (Sadowski et al. 2007; Xu et al. 2011). As a non-CE technique, the TOF methods are widely employed; however, TOF MRA is limited to single-frame static images and does not allow for hemodynamic assessment (Bi et al. 2010; Sadowski et al. 2007).

To overcome these shortcomings, non-CE 4DMRA with an arterial spin labeling technique has been developed and its use is reported to be feasible in the intracranial area (Bi et al. 2010; Xu et al. 2011; Kim 1995; Yu et al. 2012; Tan et al. 2010; Warmuth et al. 2005; Yan et al. 2010; Jahng et al. 2014). Non-CE 4DMRA employs the use of magnetically-labeled arterial blood as an endogenous contrast agent, which allows for simultaneous visualization of the vascular architecture and dynamic filling of blood in 3D (Bi et al. 2010; Xu et al. 2011; Kim 1995; Yu et al. 2012; Tan et al. 2010; Warmuth et al. 2005; Yan et al. 2010; Jahng et al. 2014). Moreover, the procedure affords 10–20-fold higher temporal and spatial resolution than that of CE 4DMRA (Yan et al. 2010). Furthermore, unlike the conventional CE 4DMRA, non-CE 4DMRA can be easily repeated (Bi et al. 2010; Xu et al. 2011; Kim 1995; Yu et al. 2012; Tan et al. 2010; Warmuth et al. 2005; Yan et al. 2010; Jahng et al. 2014).

Most studies of non-CE 4DMRA have assessed its use for the evaluation of fast-flow vascular lesions such as arteriovenous malformation (Xu et al. 2011; Kim 1995; Tan et al. 2010); its utility for the assessment of non-vascular lesions (e.g., tumors) has not been reported. Moreover, its use for the assessment of carotid lesions has not been reported (Warmuth et al. 2005; Yan et al. 2010).

In this study, we investigated the feasibility of the use of the non-CE 4DMRA technique for the evaluation of carotid vessel structure and hemodynamic characteristics of HNTs.

## Methods

### Patients

Fifteen consecutive adult patients [nine men and six women; mean age: 59.0 years (age range 28–78 years)] with HNTs were evaluated. The clinical diagnosis was as follows: 10 squamous cell carcinomas [base of the tongue (4), tonsil (1), maxillary sinus (3), salivary duct (1), and nasal cavity (1)], three carotid body paragangliomas, one pleomorphic adenoma, and one nasal cavity melanoma. All 15 patients underwent MRI, both non-CE and CE 4DMRA, between May 2011 and February 2013. Six patients (Pt. 1–6) also underwent conventional DSA. DSA was performed 2–21 (mean 8.8) days after CE 4DMRA without interval intervention. In four patients (Pt. 2, 5, 9, 15) TOF MRA was acquired at the same examination with non-CE and CE 4DMRA. The study was approved by the Ethical Board of our hospital and informed consent was obtained from all patients.

### Imaging

All patients were examined on a 3T scanner (MAGNETOM Trio, A Tim System, Siemens AG, Germany). A 12-channel head coil and 4-channel neck coil was used for signal reception.

#### Non-CE 4DMRA

Non-CE 4DMRA scans were performed according to the routine protocol for non-contrast scanning protocol at our institution. This includes localization, a T1-weighted spin echo sequence, a T2-weighted turbo spin echo sequence, and a low resolution TOF MRA for planning the orientation and position of non-CE and CE 4DMRA. Non-CE 4DMRA measurements were performed employing a previously developed 4D sequence with a flow-sensitive alternating inversion recovery scheme for spin tagging blood labeling (Bi et al. 2010; Kim 1995). Slice selective (label) or nonselective (control) inversion recovery signals were continuously sampled by a 3D true FISP, and the difference of the two acquisitions provided 4D dynamic MRA signals (Sadowski et al. 2007) (Bi et al. 2010). To minimize the effects of cardiac pulsation on dynamic MRA images, cardiac-gating was performed. Depending on the cardiac cycle and the acquisition window, 10–28 phases of non-CE 4DMRA images with an interval of trigger time of 42.5–50 ms were acquired within approximately 10 min. Imaging parameters included: TR/TE = 48.1/1.8; mean voxel size = 0.92 × 0.91 × 1.35 mm^3^; 64–104 slices per slab.

A variable (20°–45°) flip angle approach was employed to improve the visualization of late-filling vascular regions (Speier 2010), with True FISP readout and pulse trigger. Automated subtraction of non-CE 4DMRA images with nonselective and selective labeling, as well as MIP images of subtracted images in three orthogonal directions were performed inline as part of the reconstruction routine.

#### CE 4DMRA; time-resolved CE 3DMRA

All 15 patients also underwent CE 4DMRA (time-resolved imaging with stochastic trajectories) with a temporal resolution of 2.02–3.16 s with interpolation (1.02–1.58 s), with 52–92 sequential non-breath-hold datasets obtained.

A 0.2 mL/BW bolus of gadopentetate dimeglumine (Gd-DTPA, Magnevist; Bayer, Wayne, NJ) was administered at a rate of 0.7–1.5 mL/s, followed by a 30–40-mL saline flush with an automated power injector. Imaging for CE 4DMRA was started 0–10 s after injection of the contrast agent. Imaging parameters were the following: TR/TE, 3.3/1.3; flip angle, 25°; rectangular field of view (rFOV), 225–362 × 350–400 mm; matrix, 202–248 × 399–448; slice thickness, 1.35–1.6 mm; acceleration factor, 2 or 3. During acquisition of CE 4DMRA dataset, the user may select the percentage of the center of k-space (A), compared with the entire k-space volume that is sampled with every acquisition (controlling image contrast), and the percentage of data points in the periphery of k-space (B) sampled per acquisition, with subsequent savings in acquisition time, at the expense of fine detail (Lim et al. 2008). Based on a previous report (Lim et al. 2008), values of A = 10 % and B = 15 % were used in this series.

Subsequent to data acquisition, automated subtraction images with precontrast and postcontrast and MIP images of subtracted images in three orthogonal directions were performed inline as part of the reconstruction routine. Lastly, post contrast T1-weighted spin echo images were acquired.

### Image analysis

#### Qualitative evaluation

Two radiologists independently assessed the non-CE and CE 4DMRAs for overall image quality, visibility of vessel segments, and main arterial tumor feeders.

For the evaluation, MIP images of subtracted images in three orthogonal views (anteroposterior, lateral, and axial views) and source images on commercially available DICOM viewer (ClearCanvas workstation, ClearCanvas Inc. Toronto, ON, Canada) were used.

#### Overall image quality; 1–3

**Fig. 1:**
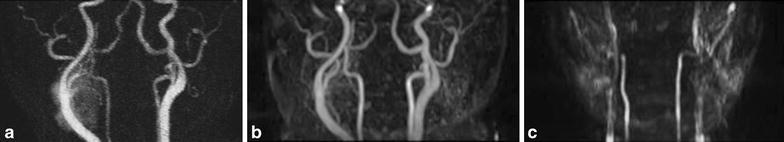
Representative images for overall image quality grade 3 (**a**), 2 (**b**), and 1 (**c**). Image **a** is a selected maximum intensity coronal projection image acquired during coronal contrast-enhanced 4DMRA, and **b** is a selected maximum intensity coronal projection image acquired during non-contrast-enhanced 4DMRA in a 43-year-old woman with carotid body paraganglioma. Image **c** is a selected maximum intensity coronal projection image acquired during non-contrast-enhanced 4DMRA in a 64-year-old man with tongue squamous cell carcinoma. *4DMRA* Four-dimensional magnetic resonance angiography

Overall image quality was evaluated on a scale of 1–3 (grade 1; insufficient, poorly visible with substantial blurring and/or artifacts; grade 2; fair, good signal and minimal blurring and/or artifacts [sufficient image quality for diagnosis]; and grade 3, excellent image quality and sharply defined borders [information was of diagnostic quality]). Sample images are shown on Fig. 1. Artifacts such as motion artifact, which hinder interpretation, were recorded.

#### Visibility of vessel segments; 0, 1

**Fig. 2:**
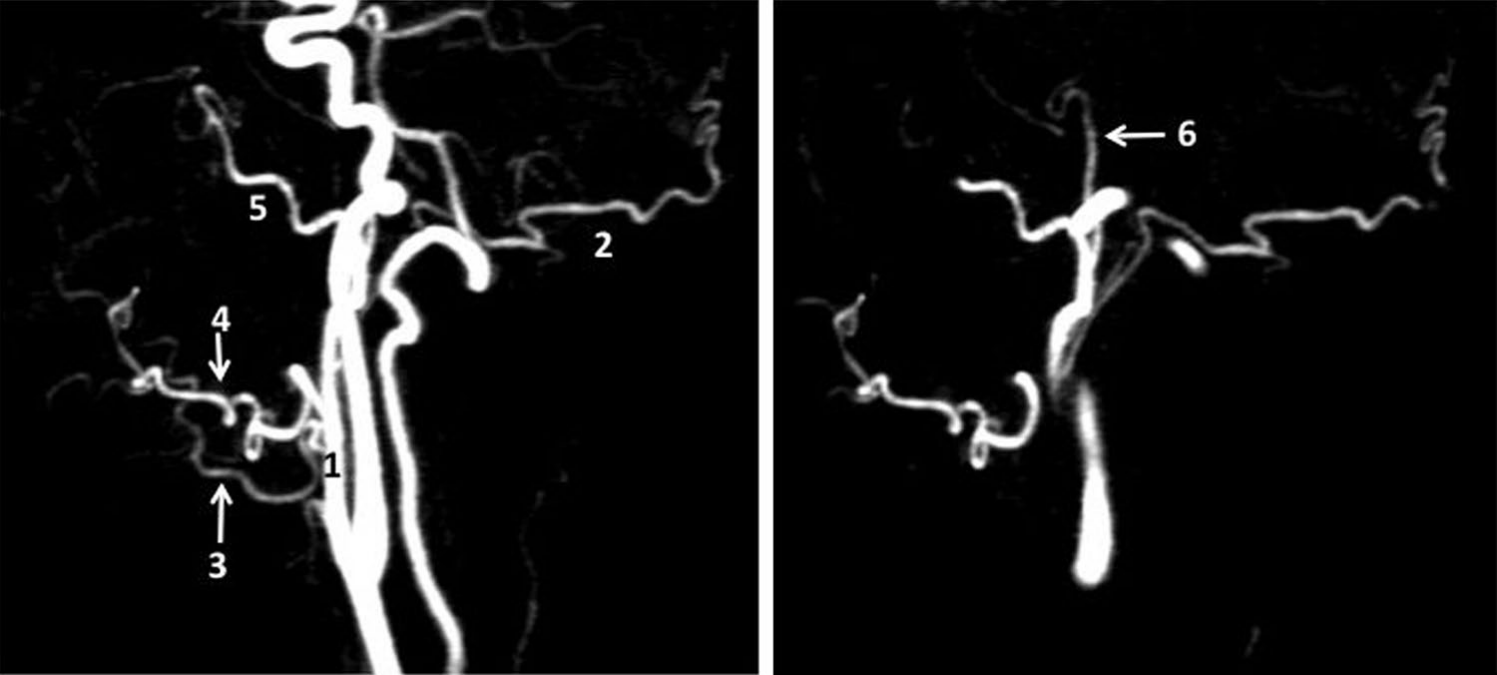
Maximum intensity lateral projection 40-mm thick images acquired during sagittal contrast-enhanced 4DMRA in a 63-year-old man with histopathologically confirmed maxillary sinus squamous cell carcinoma showing branches of external carotid artery. *1* External carotid artery, *2* occipital artery, *3* lingual artery, *4* facial artery, *5* internal maxillary artery, and *6* superficial temporal artery. *4DMRA* Four-dimensional magnetic resonance angiography

Visibility of external carotid artery system was evaluated on a binary scale, 0 or 1 (0; not visible, 1; visible). Each external carotid artery system was divided into 6 vascular segments: the main external carotid trunk and its branches, occipital artery, lingual artery, facial artery, maxillary artery, and superficial temporal artery (Fig. 2). All non-CE 4DMRA images were acquired on both sides of carotid arteries.

In patients 1, 2, 7, and 8, who underwent CE 4DMRA of hemilateral (i.e., arteries ipsilateral to the tumor) carotid vessels, we evaluated the hemilateral carotid vessels. In the other 11 patients, who underwent CE 4DMRA of bilateral carotid vessels, bilateral carotid vessels were evaluated. Therefore, six vessel segments in hemilateral carotid vessels in patients 1, 2, 7, and 8, and 12 segments in bilateral carotid vessels (six vessel segments in each side) in the other 11 patients were evaluated. Overall, a total of 156 vessel segments were evaluated in this study.

#### Main arterial tumor feeders

We defined a vessel as a tumor feeder when it entered the tumor and lost its flow signal on the consecutive slices in the tumor (van den Berg et al. 2000). On identification of two or more potential arterial tumor feeders, the largest and second largest arteries were considered as main arterial tumor feeders. To identify the tumors, we referred to the non-CE routine T2-weighted turbo spin echo images, if necessary.

Subsequent to the independent review of the visibility of vascular segments and main arterial tumor feeders, the two observers jointly reviewed the images to arrive at a consensus. Inter-observer and inter-modality agreements were calculated. The reference standard was a consensus reading by both radiologists of non-CE and CE 4DMRA, and also DSA correlation and/or TOF MRA, where available.

#### Hemodynamic evaluation

For hemodynamic evaluation, the MIP images of CE and non-CE 4DMRA (DICOM format) were transferred onto a personal computer and analyzed using Osirix (Version 5.6, OsiriX Foundation, Geneva, Switzerland; http://www.osirix-viewer.com/) software. One radiologist measured signal intensities at the ROIs determined on MIP images within the tumor on both CE and non-CE 4DMRA, and on CE 4DMRA also in the ipsilateral carotid artery bulb. To place the ROIs within the tumor, T2-weighted turbo spin echo images and pre- and post-CE T1-weighted spin echo images were referred to. Once an ROI was manually placed on a phase, the same ROIs were automatically placed onto the subsequent dynamic MIP images by the Osirix software. The signal intensity within the ROIs was automatically calculated and signal-intensity-over-time (SIT) curves prepared.

The time-to-peak signal intensity of the tumor was determined on SIT curves of both non-CE and CE 4DMRAs. On CE 4DMRA, time-to-peak of the ROIs in carotid bulb were also determined, and the duration between the time-to-peak of the tumor and of the carotid bulb was defined as ΔTP. The type of vascularity on CE 4DMRA was used as a reference standard for hemodynamic evaluation of non-CE 4DMRA. In accordance to Michaely et al. (Michaely et al. 2007), the tumors were labeled as arterial/hypervascularized if ΔTP was 0–4.6 s.

### Statistical analysis

All statistic assessments were two-sided and evaluated at a 0.05 level of significance. Inter-observer agreement for each scanning sequence was assessed using the Cohen’s kappa coefficient. The guidelines proposed by Landis and Koch were used for interpretation of the kappa coefficient. Kappa coefficients of <0.00 indicated poor; 0.00–0.20, slight; 0.21–0.40, fair; 0.41–0.60, moderate; 0.61–0.80, substantial; and 0.81–1.00, excellent or almost perfect agreement. The sensitivity and specificity of each sequence was determined by consensus, based on reference data. The binomial test was used for comparison between the sensitivity of two scanning sequences. All statistical analyses were performed using the Statistical Package for the Social Sciences (SPSS) 19.0 for Windows (SPSS Inc., Chicago, IL, USA).

## Results

### Qualitative evaluation

**Fig. 3:**
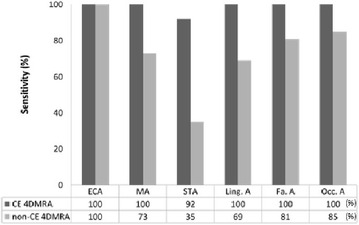
Sensitivity of CE- and non-CE 4DMRA for visualization of cervical arteries based on consensus reading. *ECA* External carotid artery, *MA* maxillary artery, *STA* superficial temporal artery, *Ling. A* lingual artery, *Fa. A* facial artery, *Occ. A* occipital artery, *CE 4DMRA* contrast enhanced four-dimensional magnetic resonance angiography, *non*-*CE 4DMRA* non-contrast enhanced 4DMRA

Non-CE 4DMRA was inferior to CE 4DMRA with respect to the overall image quality, and visualization of vessel segments and main arterial tumor feeders. Results of the vessel segment visualization on non-CE- and CE 4DMRA in the 15 patients are summarized in Fig. 3. Table 1 shows the main arterial tumor feeders delineated on non-CE and CE 4DMRA in the 15 patients.

**Table 1:** Number of ‘visible’ of feeder arteries as determined by CE 4DMRA, non-CE 4DMRA, and reference images

	Reference	CE4DMRA				non-CE4DMRA			
		Consensus reading	Observer		Sensitivity	Consensus reading	Observer		Sensitivity
			1	2			1	2	
ECA	3	3	4	3	19/20 (95 %, 95 % CI; 0.75–1.00)	2	2	2	4/20 (20 %, 95 % CI; 0.06–0.44)
MA	5	4	4	4		0	0	0	
Ling. A	4	4	5	4		0	2	0	
Fa. A	4	4	7	4		0	2	0	
As. Pha. A	3	3	1	3		2	1	2	
Total	20	19	23	19		4	7	4	

*ECA* External carotid artery, *MA* maxillary artery, *Ling. A* lingual artery, *Fa. A* facial artery, *As. Pha. A* ascending pharyngeal artery, *CE 4D MRA* contrast-enhanced 4D MRA, *non*-*CE 4D MRA* non contrast-enhanced 4D MRA

#### Overall image quality

**Fig. 4:**
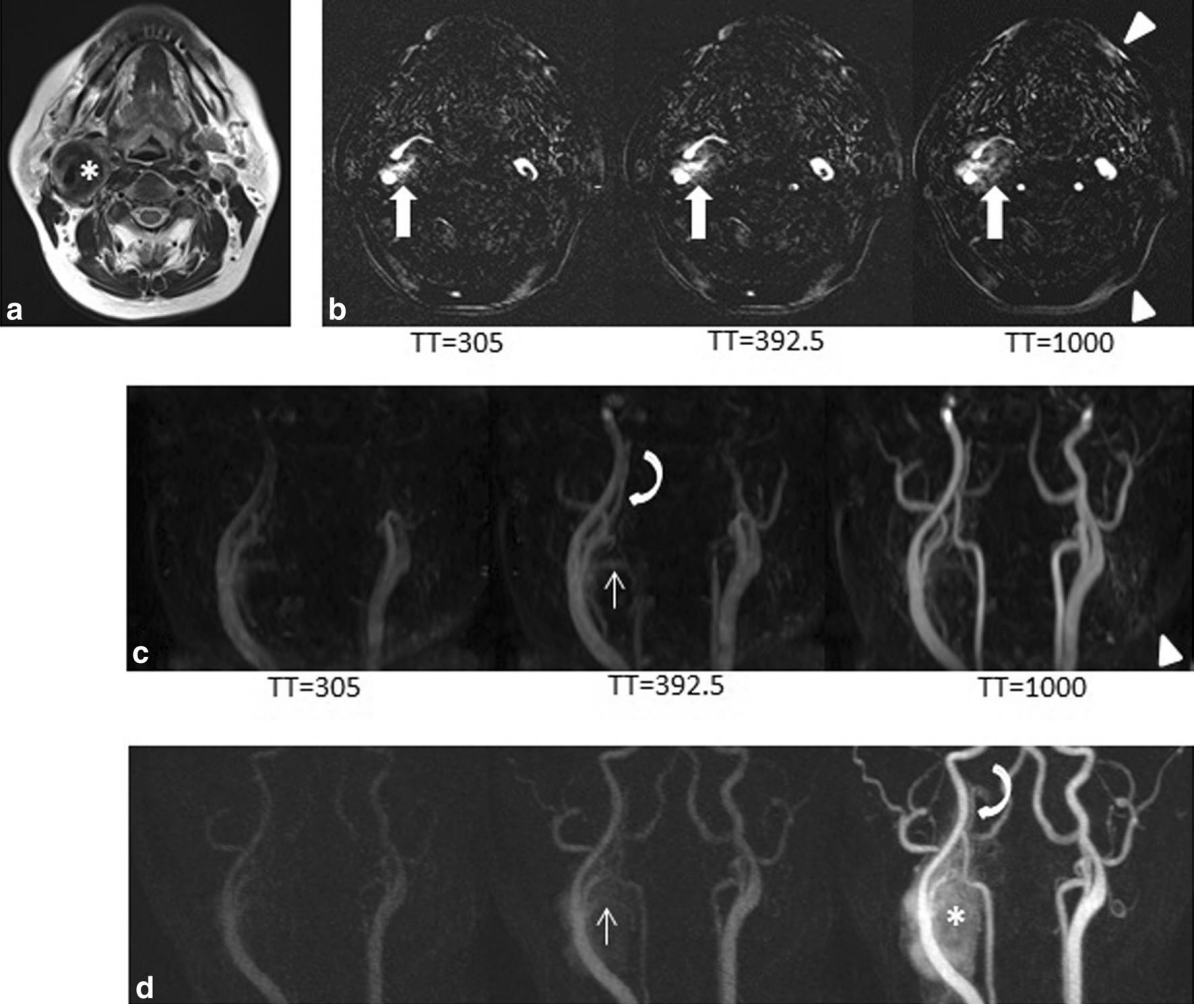
Images of a 43-year-old woman with carotid body paraganglioma. **a** Axial T2-weighted MR image showing a hypointense mass in the right carotid space (*asterisk*); **b** Selected series of subtracted source images on non-CE 4DMRA. Chronological signal increase corresponding to the tumor is indicated by *arrows*. High signals corresponding to soft tissue are indicated by *arrowheads*. **c** Selected series of anteroposterior MIP images on non-CE 4DMRA. The arterial feeders (*arrow*) derived from the right ECA and enlarged rt. ascending pharyngeal artery (*curved arrow*) are shown. High signals corresponding to soft tissue are noted (*arrowheads*). **d** Selected series of anteroposterior MIP images on CE 4DMRA. Arterial tumor feeders are derived from the right ECA (*arrow*) and enlarged right ascending pharyngeal artery (*curved arrow*). Tumor stain (*asterisk*) is seen in the early arterial phase. *TT* Trigger time, *ECA* external carotid artery, *CE 4DMRA* contrast enhanced four-dimensional magnetic resonance angiography, *non*-*CE 4DMRA* non-contrast enhanced 4DMRA

Average overall quality of non-CE and CE 4DMRA was 2.9 and 1.6, respectively. The overall image quality of CE 4DMRA was significantly higher than that of non-CE 4DMRA (CE 4DMRA: good, 13 [86.7 %]; fair, 2 [13.3 %]; insufficient, 0 [0.0 %]. Non-CE MRA: good, 0 [0.0 %]; fair, 9 [60.0 %]; insufficient, 6 [40.0 %]; *p* < 0.001). Signal of some static structures was noted on non-CE 4DMRA in 14 of 15 patients (Fig. 4b, c), while it was not noted on CE 4DMRA (Fig. 4d).

#### Visibility of vessel segments

On CE 4DMRA, 154 out of 156 vessel segments (99 %) were visualized, while only 115 vessel segments (75 %) were visualized on non-CE 4DMRA in consensus reading (Fig. 3). The depiction ratio for non-CE 4DMRA was significantly lower than that for CE 4DMRA (*p* < 0.001). Inter-observer repeatability was good with *κ* = 0.62 [Standard error (SE) = 0.07; *p* < 0.001] and moderate with *κ* = 0.56 (SE = 0.23, *p* < 0.001) for CE and non-CE 4DMRA, respectively.

#### Main arterial tumor feeders

In a reference reading, 20 main arterial tumor feeders (19 on CE 4DMRA and four on non-CE 4DMRA) were identified (Table 1). These arterial tumor feeders coincided with the feeding arteries identified on reference readings. The sensitivities of CE and non-CE 4DMRA were 0.95 and 0.20, respectively, while both modalities showed a specificity of 1.00. The specificity of CE 4DMRA was significantly higher than that of non-CE 4DMRA (*p* < 0.001). The inter-observer repeatability for CE and non-CE 4DMRA was good {*κ* = 0.70 [standard error (SE) = 0.08; *p* < 0.001]} and moderate [*κ* = 0.54 (SE = 0.18), *p* < 0.001}, respectively.

### Hemodynamic evaluation

**Fig. 5:**
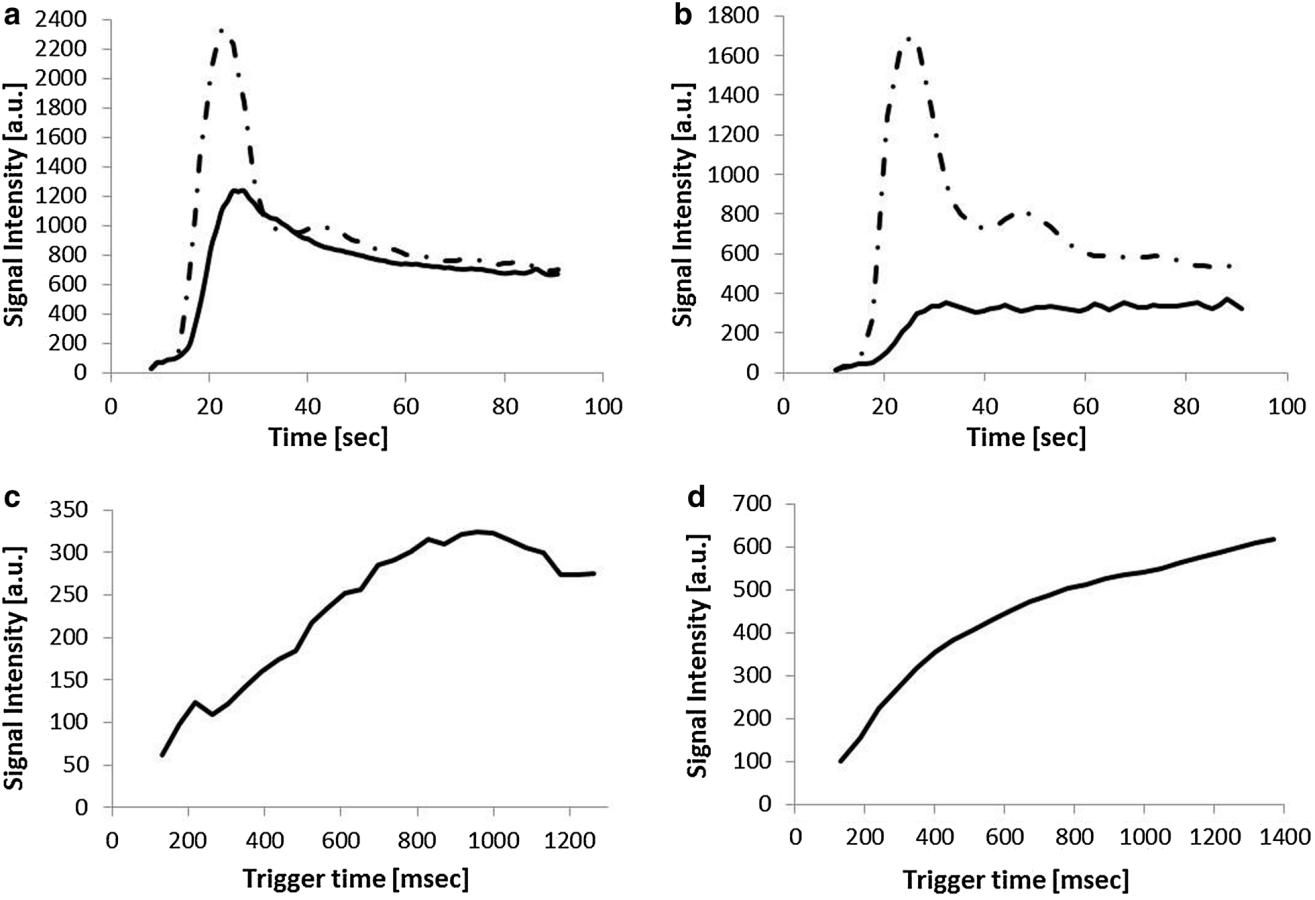
Hemodynamic assessment of signal intensity with ROIs placed in the tumor (*solid line* in **a**–**d**) and the carotid artery (*broken line* in **a**, **b**) in patients with paraganglioma (43-year-old woman, **a**, **c**) and in patients with SCC of the base of tongue (64-year-old man, **b**, **d**). The *upper row* (**a**, **b**) shows the signal-intensity-over time curves from CE 4DMRA and those in the *bottom row* (**c**, **d**) are from non-CE 4DMRA. *a.u.* Arbitary unit, *ROI* region of interest, *CE 4DMRA* contrast enhanced four-dimensional magnetic resonance angiography, *non*-*CE 4DMRA* non-contrast enhanced 4DMRA

In three patients (patient nos. 9, 10, and 15), SIT curves from CE 4DMRA were consistent with an arterial/hypervascularized pattern [ΔTP < 4.4 s (mean 2.5 s)] (Fig. 5a). These three tumors were all paragangliomas. The other tumors, i.e., ten squamous cell carcinomas, one pleomorphic adenoma, and one nasal cavity melanoma showed larger ΔTP (5.3–57 s; mean 16.6) (Fig. 5b). The SIT curves from CE 4DMRA were consistent with those reported by Michaery et al. (Michaely et al. 2007). All three arterial/hypervascular tumors classified on CE 4DMRA exhibited a distinct peak followed by signal decrease (washout) on non-CE 4DMRA (Fig. 5c), while the others showed a gradual signal increase and no clear decrease (Fig. 5d).

## Discussion

The results of this study suggest that as a completely noninvasive method, non-CE 4DMRA is an effective aid in the evaluation of vascular architecture and allows for the identification of hypervascular tumors. However, poor image quality and the short coverage of the time window are two major shortcomings.

### Qualitative evaluation

Compared with CE 4DMRA, lower overall image quality and poor visualization for main tumor feeding arteries and some external carotid branches were noted on non-CE 4DMRA.

The poor image quality of non-CE 4DMRA may be attributable to the low signal-to-noise ratio, motion artifacts, and off-resonance artifacts, which are the major drawbacks of non-CE 4DMRA (Bi et al. 2010).

The signal-to-noise of non-CE 4DMRA is relatively low since the difference between slice selective (label) and nonselective (control) inversion recovery signals is <1 % (Bi et al. 2010; Jahng et al. 2014). The signal depends on the blood T1 relaxation time and labeling decay after a delay time (Jahng et al. 2014). During the inflow, both inverted and non-inverted blood evolves towards the steady state, so that the difference between both decreases over time and vessels filling late in the inflow period are captured with lower signal (Bi et al. 2010; Speier 2010). We employed variable (20°–45°) flip angles to retain the contrast (Speier 2010); however, the image quality was still poor.

Therefore, we speculate that the poor image quality was mainly caused by the latter two reasons, namely motion artifacts and off-resonance artifacts. In contrast to the intracranial area, the head and neck area is much more prone to motion and off-resonance artifacts. Pulsation of carotid arteries, breathing, and swallowing are unavoidable sources of significant motion artifacts (Boussel et al. 2006). The interfaces of tissues with different magnetic susceptibility, such as those between air, bone, and tissue, are responsible for off-resonance artifacts (Boussel et al. 2006). Off-resonance artifacts can be offset between inverted and non-inverted series, but, due to the interference by motions mentioned above, it is not possible to offset off-resonance artifacts.

In our series, non-CE 4DMRA visualized some ECA branches such as the maxillary, lingual, facial, and occipital arteries with a good sensitivity. These arteries frequently play the role of main arterial feeders for HNTs which requires endovascular procedures such as embolization or super-selective local chemotherapy (Kashiwagi et al. 2010; Uehara et al. 2010; Nakasato et al. 2000). Although we did not evaluate visualization of ascending pharyngeal artery, some enlarged ascending pharyngeal arteries were identified as arterial feeders on non-CE 4DMRA. The main arterial feeders are largely derived from the maxillary artery and facial artery for maxillary SCC (Kashiwagi et al. 2010), lingual, maxillary, and facial arteries for oral SCCs (Uehara et al. 2010; Nakasato et al. 2000), and the ascending pharyngeal and occipital artery for paragangliomas (van den Berg et al. 2000). Thus, non-CE 4DMRA may have a role to play in endovascular procedures. In future, if we could avoid the artifacts mentioned above, with its higher temporal and spatial resolutions, non-CE 4DMRA would enable us to identify the feeding arteries better than would 4D CEMRA, which has been reported to be insufficiently reliable enough for identifying feeding arteries (Nishimura et al. 2012).

In our series, sensitivity of main tumor vessels on CE 4DMRA was 95 % with good (κ = 0.70) inter-observer agreement, which is better than that reported by Nishimura et al. (2012). They evaluated CE 4DMRA of brain tumors and HNTs qualitatively in a series comprising of 15 patients, of which seven had HNTs [buccal cancer (2), buccal hemangioma (2), juvenile angiofibroma (1), metastatic bone tumor from hepatocellular carcinoma (1), and nasal cavity carcinoma (1)] (Nishimura et al. 2012). We used source images of CE 4DMRA besides MIPs, while they did not use source images. This explains the difference in the results, as the use of source images increases the sensitivity for detection of tumor feeders. In clinical settings, use of source images may not be practical because of the data volume of whole 4DMRA. Differences the in study population may also have affected the results.

### Hemodynamic evaluation

Michaery et al. reported that CE 4DMRA can differentiate hyper vascular tumor from others (Michaely et al. 2007). On CE 4DMRA, all paragangliomas showed arterial/hypervascularized pattern, i.e., peak enhancement in <4.6 s after that of the ipsilateral carotid bulb, while SCCs, scar tissue, and hyperplasia of the nasopharyngeal lymphatic tissue showed significantly reduced signal intensity, with a weak or no peak visible (Michaely et al. 2007; Lazzaro et al. 2011; van den Berg et al. 2000).

In our series of non-CE 4DMRA, all paragangliomas which showed arterial/hypervascularized pattern, showed peak signal on SIT curves, while other tumors showed no clear peak. This suggests that non-CE 4DMRA can differentiate paragangliomas, in which pre-operative embolization is indicated (Michaely et al. 2007), from other tumors.

The source of signals is different between CE and non-CE 4DMRA. CE 4DMRA uses gadopentetate dimeglumine as an exogenous contrast medium. Extracellular contrast medium diffuses from the blood into the extravascular extracellular space at a rate determined by tissue perfusion, the permeability of the capillaries, and their surface area. Shortening of the T1 relaxation rate caused by the contrast medium is the mechanism of tissue enhancement (so-called T1 or relaxivity-based methods) (Jahng et al. 2014). The time window captures tumor stain at the early and late arterial, parenchymal, and venous phases (Nishimura et al. 2012). On the other hand, non-CE 4DMRA uses magnetically labeled inflowing arterial blood as an endogenous contrast agent. The signal depends on the blood T1 relaxation time and labeling decay after a delay time (Jahng et al. 2014). After the tagging, tagged blood flows into the imaging slab following a transit delay to allow these tagged spins to enter the imaging plane and exchange with tissue (Jahng et al. 2014). This signal nature shortens the time window for non-CE 4DMRA (approximately 1–2 s) (Yan et al. 2010), and the detectable vessel length depends on the flow velocity and the delay time after spin labeling (Bi et al. 2010; Speier 2010).

In our series, it seems that the limited time window for non-CE 4DMRA allowed only paragangliomas to show signal peak. In other tumors that need longer transit time i.e., located in longer and/or slower inflowing feeder, the time window may not be wide enough to allow the tagged blood to show tumor signal peak. Non-CE 4DMRA may help identify arterial/hypervascularized tumors, and in particular, paraganglioma, which is a candidate for pre-operative embolization (Lazzaro et al. 2011). Further studies with a greater variety of hypervascularized tumors such as juvenile nasopharyngeal angiofibroma are desirable. In future, techniques for maintaining signal contrast between non-inverted blood and inverted static tissues, and thus, a longer time window would enable us to evaluate tumors with different hemodynamic profiles such as slower inflow blood.

There are some limitations in our study. First, though 11 of 15 patients underwent gold standard DSA in this study, four patients lacked the gold standard correlation; at our institution, management decisions are based on MRA findings, with DSA reserved for interventional management. However, most vessels were clearly visualized in an anatomically consistent pattern on CE 4DMRA, which suggests its utility for mapping of the vessels. Secondly, our data were obtained from a relatively small sample size and a limited variety of tumors. Paraganglioma was the sole type of arterial/hypervascularized tumor. Further studies with larger sample sizes and a wider variety of patients are warranted. Thirdly, we did not acquire ΔTP for non-CE 4DMRA because the signal in the carotid artery bulb could not be measured in some series because of the poor image quality, which seemed to be caused by the pulsation of carotid artery itself.

## Conclusions

This study provides information on the feasibility of non-CE 4DMRA for evaluation of vascularity of HNTs. Use of non-CE 4DMRA is feasible for assessment of vascularity of HNTs. Although it is still inferior to CE 4DMRA at present, non-CE 4DMRA may be useful in differentiating hypervascular HNTs from others. In future, with further improvements in image quality and longer coverage of time window, non-CE 4DMRA could be a noninvasive alternative to CE 4DMRA for evaluation of vascularity of HNTs. Furthermore, we can acquire both non-CE and CE 4DMRA in one MR examination. Indeed, these two techniques may complement each other and provide useful, clinically relevant information.

## Abbreviations

DSA: digital subtraction angiography
4D: four-dimensional
CE: contrast enhanced
HNTs: head and neck tumors
SCCs: squamous cell carcinomas
SIT: signal-intensity-over-time
SE: standard error
